# Transfer of immunoglobulins through the mammary endothelium and epithelium and in the local lymph node of cows during the initial response after intramammary challenge with *E. coli *endotoxin

**DOI:** 10.1186/1751-0147-50-26

**Published:** 2008-07-02

**Authors:** Karin Östensson, Shichun Lun

**Affiliations:** 1 Department of Clinical Sciences, Division of Reproduction, Faculty of Veterinary Medicine and Animal Science, Swedish University of Agricultural Sciences, Uppsala, Sweden; 2 Department of Medicine, Johns Hopkins University School of Medicine, Baltimore, USA

## Abstract

**Background:**

The first hours after antigen stimulation, interactions occur influencing the outcome of the immunological reaction. Immunoglobulins originate in blood and/or are locally synthesized. The transfer of Ig isotypes (Igs) in the udder has been studied previously but without the possibility to distinguish between the endothelium and the epithelium. The purpose of this study was to map the Ig transfer through each barrier, separately, and Ig transfer in the local lymph nodes of the bovine udder during the initial innate immune response.

**Methods:**

The content of IgG1, IgG2, IgM, IgA and albumin (BSA) was examined in peripheral/afferent mammary lymph and lymph leaving the supramammary lymph nodes, and in blood and milk before (0 h) and during 4 hours after intramammary challenge with *Esherichia coli *endotoxin in 5 cows.

**Results:**

Igs increased most rapidly in afferent lymph resulting in higher concentrations than in efferent lymph at postinfusion hour (PIH) 2, contrary to before challenge. Ig concentrations in milk were lower than in lymph; except for IgA at 0 h; and they increased more slowly. *Afferent lymph:serum *and *efferent lymph:serum *concentration ratios (CR) of Igs were similar to those of BSA but slightly lower. *Milk:afferent lymph *(M:A) CRs of each Ig, except for IgG2, showed strikingly different pattern than those of BSA. The M:A CR of IgG1, IgM and IgA were higher than that of BSA before challenge and the CR of IgA and IgG1 remained higher also thereafter. At PIH 2 there was a drop in Ig CRs, except for IgG2, in contrast to the BSA CR which gradually increased. The M:A CR of IgM and Ig A *decreased *from 0 h to PIH 4, in spite of increasing permeability.

**Conclusion:**

The transfer of Igs through the *endothelium *appeared to be merely a result of diffusion although their large molecular size may hamper the diffusion. The transfer through the *epithelium *and the Ig concentrations in milk seemed more influenced by selective mechanisms and local sources, respectively. Our observations indicate a selective mechanism in the transfer of IgG1 through the epithelium also in lactating glands, not previously shown; a local synthesis of IgA and possibly of IgM, released primarily into milk, not into tissue fluid; that IgG2 transfer through both barriers is a result of passive diffusion only and that the content of efferent lymph is strongly influenced by IgG1, IgM and IgA in the mammary tissue, brought to the lymph node by afferent lymph.

## Background

Bovine mastitis has been extensively studied but mainly as reflected in milk and circulating blood. Investigations of the reaction as it appears in the tissue, usually performed in tissue specimens, have added important information and improved the understanding of immunological reactions of the mammary gland. For studies of tissue reactions over time when repeated sampling is desirable, it appears more suitable to examine interstitial fluid that can be sampled frequently after it has entered the collecting vessels of the peripheral (afferent) lymphatic system in the tissue, through application of a semi-permanent catheter. This method was used in the present investigation parallel to examination of efferent lymph, leaving the local supramammary lymph nodes and analysis of milk. It enabled us to follow the inflammatory response along the entire pathway from the mammary milk compartment, through the interstitial space in the tissue, the afferent lymphatics and the local lymph node; a route where the immune events are initiated and of significant immunological interest. It further made it possible to separately study the transfer of various components through, on one hand the mammary endothelium and on the other hand the mammary epithelium.

Acute inflammation is the most important innate immune mechanism, by which antigens can be rapidly recognized and destroyed. During the first hours after antigen stimulation important immunological interactions occur with decisive influence on the further development and outcome of the immunological reaction. From the tissue, antigen and locally released immunological factors like immunoglobulins are rapidly transported through the afferent lymphatics to the local lymph node [1,2] which is an important site for antigen-cell and cell-cell interactions, necessary in the immune defence. Concentrations of immunoglobulins in bovine milk and afferent lymph increase shortly after antigen stimulation [3-5] and injected soluble antigen in tissue has been found to reach the local draining lymph node already within a few minutes after injection [6]. The lymph node destroys antigens, but also modulate the leukocyte and immunoglobulin output [7,8].

Soluble antibodies or immunoglobulins play important roles in the immune defence, through their opsonizing ability but also by binding and neutralizing antigens and toxins, and by preventing adherence of microbes to epithelial surfaces. Four Ig isotypes (Igs) are known to influence mammary gland defence against invading antigens: IgG1, IgG2, IgM and IgA. Igs in milk and tissue are either derived from blood through passive diffusion and/or active transport, synthesized locally, or a combination of the two. Many studies of the transfer of Igs from blood to milk under normal and inflammatory conditions have been performed [3,4,9] with the aim to identify possible local release and/or selective mechanisms influencing the transfer. However, these studies have not made it possible to investigate the transfer through each of the two barriers, the mammary endothelium and epithelium, separately.

Igs, as well as other factors, arrive to the local lymph node through two different routes; afferent lymph and blood [10]. Additionally, some of the incoming substances may be kept and other may be added by local synthesis in the node, resulting in a modulation of the output in efferent lymph. Afferent lymph is, immunologically, significantly important for the reactions in the lymph nodes through its content of antigens and immunological factors originating in the tissue. It could, however, be assumed that afferent lymph has a minor quantitative impact on efferent lymph, considering the low quantities of various components in afferent lymph, compared to blood, and its low flow rate. Information about characteristics of the transvascular transfer of different Igs in the lymph nodes and to what extent afferent lymph and blood derived factors, respectively, contribute to the content of efferent lymph is limited. This condition in the local mammary lymph nodes of cows has, to the best of our knowledge, never been examined.

The purpose of the present study was to investigate the initial phase of the innate immune response in the mammary tissue and local lymphatic system of cows, after local antigen stimulation. To experimentally induce an inflammatory reaction endotoxin from *Escherichia coli *was infused into the mammary gland. Endotoxin is considered the key factor in *E. coli *mastitis and intramammary infusion of purified endotoxin is known to initiate a pronounced acute inflammatory response in the mammary gland and local lymphatic system that can be observed already within a few hours [5,11,12]. In this paper we describe the simultaneously measured content of Igs in milk and the local mammary lymphatic compartments, and the Ig traffic through endothelium and epithelium, and in the local lymph nodes of the bovine udder. We believe this has not been described previously. The cell traffic and content of cytokines in the mammary lymph compartments and milk during this time period have previously been reported by our group [12,13].

## Methods

### Animals

Five primiparous dairy cows of the Swedish Red and White breed (SRB) were used. All cows were clinically healthy at the start of the experiment. The cows were in mid-lactation, producing approximately 20 l milk/day each. The udders of the cows were pathogen-free prior to the experiment as determined by bacteriological examination of quarter milk samples 1 week before the start of experiment. At the day before surgery, California Mastitis Test (CMT) results of left hind quarters were negative, and those of the other quarters were negative or showed the lowest degree of a positive CMT reaction ("trace") [14]. The cows had free access to water until they were brought to the surgery room but did not get any feed in the morning before the start of the surgery procedure.

### Surgical procedure

The surgical and experimental procedure was according to Lun et al. [12], and was approved by the Ethical Committee for Experimentation in Animals, Uppsala, Sweden. In short, the cows were fitted with a semi-permanent catheter in the jugular vein for blood sample collection and intravenous infusions. To enable sampling of lymph, one vessel afferent and one vessel efferent to the left supramammary lymph node was catheterized. Intubation was performed, and anaesthesia was maintained with halothane in oxygen and nitrous oxide. A peripheral lymph vessel in the udder tissue of the left hind quarter was catheterized according to a surgical procedure described by Obel et al. [15]. The efferent vessel was catheterized just before its entry into the inguinal canal, according to Kottman et al. [16].

A number of vital functions were checked and registered from the start of the anaesthesia until the end of the experiment to control the general condition and hydration of the animals: Arterial blood pressure, heart rate, electrocardiography, blood gas kinetics and pH in arterial blood, total and differential leukocyte counts and hematocrit (to monitor eventual dehydration). Intravenous fluid therapy (40 ml per kg bodyweight) with a buffered hydration solution (Ringer-acetat^®^, Pharmacia & Upjohn, Stockholm, Sweden) was applied during the anaesthesia. To ensure adequate ventilation and to maintain an appropriate arterial carbon dioxide pressure (5–6 kPa), the cows were mechanically ventilated. The animals were euthanized, while still under general anaesthesia.

### Experimental design

Approximately 2 h after completion of the cannulation (0 h), the first set of samples (milk, blood, afferent and efferent lymph) were collected after which 50 μg of *Esherichia coli *O type 055:B5 endotoxin (Sigma Chemical Co., St. Louis, MO) in 10 ml phosphate-buffered saline solution (PBSS) was infused into the left hind quarter through the teat canal [12]. The dose of endotoxin used was determined according to previous studies showing that such a dose is capable of inducing a mild inflammatory reaction [3,11,17]. Milk, blood and lymph samples were also collected at post-infusion hour (PIH) 2 and 4.

### Lymph, milk and blood sampling

Sampling was performed as described by Lun et al. [12]. Stripping milk samples (5 ml) were collected from the left hindquarter after it had been emptied by hand milking. At each sampling also 10 ml of blood from the jugular vein and 5 ml of afferent and efferent lymph, respectively, were sampled. Blood and lymph were collected in plain tubes for analysis of bovine serum albumin (BSA) and Ig isotype concentrations, and in EDTA tubes (Venoject^®^, Terumo Europe N.V., Leuven, Belgium) for analysis of total and differential leukocyte counts. The tubes were immediately centrifuged and the supernatant was collected and analysed. The samples used for analysis of BSA and Igs were stored in -20°C until analysed.

### Immunoglobulin assay

IgG1, IgG2, IgM, IgA and BSA concentrations (mg/ml) were determined by Radial Immunodiffusion (RID) Kits (BINDARID™, the Binding Site Ltd, Birmingham, UK). High, medium and low calibrators were used and samples were diluted accordingly. The concentrations of Igs and BSA were calculated by linear regression.

### Leukocyte counts

Total and differential leukocyte counts in *milk *were determined using direct light microscopy according to the reference method for milk (IDF standard IDF 148-1/ISO/DIS 13366-1). *Lymph *samples were treated according to Lun et al. [12]. Lymph was mixed 2:1 with PBSS, and centrifuged for 10 min. at 500 g. The supernatant was removed and the cell pellet was resuspended up to 1 ml with PBSS and 1 drop of homologous serum. Strips for total leukocyte counts were prepared using the cell suspension according to the procedure for milk. Smears for differential leukocyte counting were prepared and stained using the conventional May-Grünewald-Giemsa method. Lymph leukocyte counts were determined using direct light microscopy. *Blood *samples were analysed fresh for total and differential leukocyte counts according to the standard procedure used at the laboratory of the Department of Clinical Chemistry, Swedish University of Agricultural Sciences, Uppsala, Sweden.

### Statistical analysis

The statistical analyses were performed using the SAS-program (SAS Inst. Inc., Cary, NC). Analysis of variance (PROC MIXED) was applied to the data, according to two different models. 1. The recorded concentrations (of IgG1, IgG2, IgM, IgA and BSA) were analysed according to a statistical model including the fixed effects of sampling occasion, fluid and the interaction between sampling occasion and fluid. The statistical model also included the random effect of animal. 2. Ratios between concentrations in milk, lymph and blood serum (*afferent lymph:serum, efferent lymph:serum and milk:afferent lymph*) were constructed. These ratios were analysed according to a statistical model including the fixed effects of sampling occasion, parameter (IgG1, IgG2, IgM, IgA and BSA) and the interaction between sampling occasion and parameter. The statistical model also included the random effect of animal. Least-square means were estimated and compared using t-test.

## Results

### Clinical data and leukocyte counts

During the entire experimental period, the registrations of all vital body functions checked showed that the functions remained stable and with values within normal range. Visible signs of acute clinical mastitis were observed in the endotoxin-infused quarter within the first hour after infusion and were pronounced at PIH 2. Both afferent and efferent lymph flow rate increased gradually 8-fold after endotoxin infusion.

Detailed information on changes in leukocyte counts in milk, lymph and blood is reported by Lun et al. [12]. In short, the total leukocyte concentration (log10/ml) in milk increased slightly but not significantly at PIH 4 (0 h, 5.30 ± 0.80; 4 h, 5.96 ± 0.25). The proportion of neutrophils in milk increased significantly (p < 0.05) at PIH 4 (0 h, 7 ± 1%; 4 h, 30 ± 8%). In afferent lymph, the total leukocyte concentration (log10/ml) increased (p < 0.05; 0 h, 5.63 ± 0.17; 4 h, 6.23 ± 0.14), while the concentration (log10/ml) in efferent lymph decreased (p < 0.05) at PIH 4 (0 h, 6.27 ± 0.10; 4 h, 5.95 ± 0.09). In afferent lymph, lymphocytes were the predominant cell type before infusion while neutrophils dominated both at 2 and 4 h after endotoxin infusion (0 h, 6 ± 1%; 2 h, 55 ± 15%; 4 h, 79 ± 4%). In efferent lymph, lymphocytes dominated throughout the study. However, the proportion of neutrophils was increased (p < 0.05) at PIH 4 (0 h, 0%; 4 h 17 ± 7%).

### Concentrations of immunoglobulins and BSA

**Figure 1:**
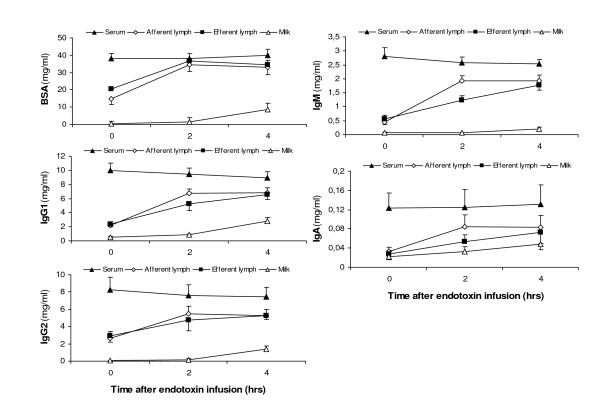
**Concentrations of immunoglobulin isotypes and bovine serum albumin (BSA) in afferent and efferent lymph, milk and blood serum, before and after intramammary infusion of 50 μg of *Escherichia coli *endotoxin**. Data are expressed as LS-mean ± SEM. Time 0 refers to samples collected before the endotoxin infusion.

Concentrations of Igs and BSA were lower in milk than in lymph and BSA was lower in afferent than in efferent lymph, at all time points. The concentration of each Ig was similar in afferent and efferent lymph before the challenge and at PIH 4, respectively, while at PIH 2 concentrations were highest in afferent lymph. Results are shown in Figure 1. Before endotoxin infusion, the concentrations (mg/ml) of IgG1, IgG2, IgM, IgA and BSA in *milk *were 0.56, 0.04, 0.06, 0.030 and 0.32; in *afferent lymph *2.22, 2.61, 0.45, 0.032 and 14.65; in *efferent lymph *2.39, 2.95, 0.57, 0.027 and 20.45; and in blood serum 9.98, 8.25, 2.81, 0.123 and 38.09, respectively. At PIH 2 the corresponding figures were in *milk *0.84, 0.17, 0.06, 0.033 and 1.55; in *afferent lymph *6.74, 5.48, 1.92, 0.085 and 34.37; in *efferent lymph *5.27, 4.76, 1.24, 0.053 and 36.69; and in blood serum 9.43, 7.62, 2.57, 0.125 and 37.95, respectively.

The concentration of all Igs and BSA increased most rapidly in *afferent lymph *where significantly increased concentrations of IgG1 (p < 0.001), IgG2 (p < 0.001), IgM (p < 0.001), Ig A (< 0.05) and BSA (p < 0.01) were observed already from PIH 2 (Fig. 1). Also in *efferent lymph*, increased concentrations of IgG1 (p < 0.001), IgG2 (p < 0.05), IgM (p < 0.001) and BSA (p < 0.001) were seen from PIH 2, while elevated concentration of IgA was not observed until PIH 4 (p < 0.05). Between PIH 2 and PIH 4 the Ig concentrations in afferent lymph did not change, while they further increased in efferent lymph. In *milk*, increased Ig and BSA concentrations were, generally, not seen until PIH 4, when IgG1 and BSA were significantly elevated (p < 0.01 and p < 0.05, respectively) while IgG2 and IgA tended to be increased (p < 0.07 and p < 0.08, respectively). The milk concentration of IgM remained unchanged post-infusion. As expected, the concentrations of BSA and all Igs measured were highest in blood serum and they all remained unchanged after endotoxin infusion.

### Transfer of immunoglobulins and BSA

**Figure 2:**
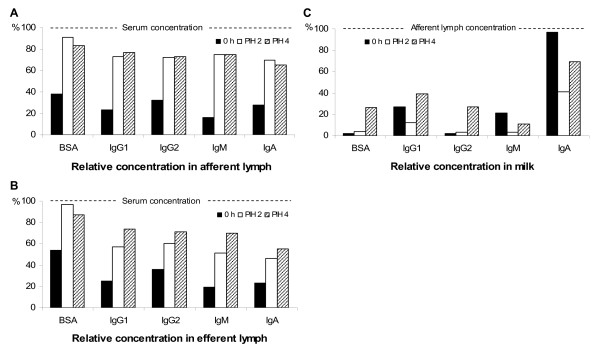
**Relative concentrations of immunoglobulin isotypes and bovine serum albumin (BSA) before and after intramammary infusion of 50 μg of *Escherichia coli *endotoxin**. Afferent lymph:serum (A), efferent lymph:serum (B) and milk:afferent lymph (C) concentration ratios express the percentage of the blood serum concentration that is simultaneously found in afferent (A) and efferent (B) lymph, respectively, depicting the transfer through the endothelium; and the percentage of the afferent lymph concentration that is simultaneously found in milk (C), depicting the transfer through the mammary endothelium. Each value represents the LS-mean. Samples were collected before endotoxin infusion (0 h) and at postinfusion hours (PIH) 2 and 4.

To a varying extent, there is a general transduction of all Igs through the endothelium and epithelium, dependent on the permeability conditions. BSA in body secretions is considered to be a result of passive diffusion only. To evaluate the influence of selective mechanisms on the transfer or the presence of local synthesis, the ratio between the Ig concentrations on each side of a barrier like the endothelium (*afferent lymph:blood serum *and *efferent lymph:blood serum*) or the epithelium (*milk:afferent lymph*) can be compared with that of BSA [18,19]. Concentration ratios (CR) of IgG1, IgG2, IgM, IgA and BSA are presented in Figure 2, showing the concentration in lymph fluid expressed as the percentage of the blood serum concentration, (Fig. 2A and Fig. 2B) and the concentration in milk expressed as the percentage of the lymph/tissue fluid concentration (Fig 2C).

The CR of all Igs at the mammary as well as the lymph node *endothelium *increased significantly (p < 0.01) during the inflammatory reaction (Fig. 2A and Fig. 2B) but the CR of IgG1, IgM and IgA, respectively, was significantly *lower *than that of BSA at 0 h and PIH 2 (p < 0.05). In the lymph node this was also true for IgG2 (p < 0.05). At PIH 4, mainly as an effect of that the CR of BSA had declined, no significant difference was observed between the endothelial CR of BSA and that of each Ig, respectively, except for IgA (p < 0.001) and IgM (p < 0.05) at the endothelium in the *lymph node*, only.

At the *epithelium*, in contrast to the endothelium, the CR at 0 h of IgG1 and IgA, respectively, was significantly *higher *(p < 0.05 and p < 0.001) and IgM tended to be higher (p = 0.07) than that of BSA (Fig. 2C). The epithelial CR of IgA remained significantly higher than that of BSA during the entire study (p < 0.001). At PIH 2, a notable drop in the CR of all Igs, except for IgG2, was observed at the mammary epithelium. The drop was highly significant for IgA (p < 0.001) and tended to be significant for IgG1 and IgM (p = 0.13 and p = 0.11). Thereafter the Ig CR increased again and at PIH 4 the CR of IgG1 was numerically higher (not statistically significant, ns), while that of IgM (ns) and IgA (p < 0.05) was still numerically lower than the 0 h value. The CR of IgG2 at the epithelium increased without any interruption from 0 h to PIH 4, and the values were almost identical to those of BSA.

## Discussion

### Pre-challenge conditions and the inflammatory response

We believe this to be the first report describing the Ig concentrations and transfer between blood, mammary lymph compartments and milk after antigen stimulation of the udder in cows. As expected, the concentrations of IgG1, IgG2, IgM, IgA and BSA increased in all fluids examined, except for blood, but with differences in their relative transfer through the endothelium and epithelium, respectively.

As reflected in the cellular response in milk and afferent lymph after the endotoxin infusion, the inflammatory response in the present study was similar to what has previously been reported [3,5,20,21]. The increase in milk SCC was less pronounced than in the studies referred to, probably related to the relatively low dose of endotoxin used [3]. The leukocyte concentration in afferent lymph and the proportion of neutrophils in milk and afferent lymph, however, increased significantly to magnitudes that are in accordance with previous reports, clearly confirming an inflammatory response.

It can be questioned whether the observed inflammatory responses really were due to the endotoxin infusion and not to the surgical trauma [22]. In the samples taken immediately before the endotoxin infusion (0 h) neutrophils comprised 7 ± 1% of leukocytes in milk, 6 ± 1% of leukocytes in afferent lymph and 0 ± 0% of leukocytes in efferent lymph. These values are in accordance with neutrophil concentrations in bovine milk and afferent and efferent lymph in the absence of inflammation [23]. Also the concentrations of different Ig isotypes and cytokines in milk and lymph before the challenge in our study [13] were on a whole in accordance with what has previously been observed in normal milk from cows [4,5,24-26] and in peripheral lymph under non-inflammatory conditions [5,18,27-29]. Thus, there was no indication of an inflammatory reaction being present in the udder tissue before the endotoxin was infused and it is likely that the observed inflammatory response was due to the endotoxin infusion and not to surgical trauma.

*BSA *is the only parameter that was slightly higher before challenge in both milk and lymph compared to most previous studies referred to. The higher milk BSA concentration was due to higher content in afferent lymph and not to increased relative transfer through the *epithelium*. This transfer was similar to previous observations in cows [5]. Inactivity has been shown to cause elevated albumin concentration in peripheral lymph [19] and is the most plausible explanation for this finding before challenge. Although the relative transfer of BSA through the *endothelium *before inflammation was slightly higher than previously shown this was, interestingly, not the case for transfer of the Igs through the same barrier, further indicating that the Ig response was not triggered before the endotoxin infusion.

The general differences between milk, and each of the two lymph fluids under normal conditions, with lower contents of BSA and Ig in milk than in afferent lymph and highest in efferent lymph agree on a whole with previously recorded data [10]. However, the information about cow mammary lymph is limited and Ig in efferent mammary lymph of cows has, to our knowledge, not been investigated previously.

### Immunoglobulin concentrations

*After endotoxin infusion *the Ig concentrations in both lymph fluids increased rapidly with significantly increased concentrations generally recorded at PIH 2. At 0 h and PIH 4, respectively, the Ig content was similar in the two lymph fluids. The most rapid increase was observed in afferent lymph, leading to that at PIH 2 the content of *IgM and IgA *in afferent lymph was significantly higher (IgM p < 0.001; IgA p < 0.05) and *IgG1 *tended to be higher (p = 0.055), than in efferent lymph (Fig. 1). If the source of these Igs had been blood only, the increase we observed should have occurred equally rapid in the two lymph fluids, similar to the pattern of the BSA concentrations (Fig. 1). However, increased concentrations of IgA, IgM and IgG1 were observed earlier in afferent than in efferent lymph. This indicates a local increase of these Igs in tissue that was measurable in afferent lymph at PIH 2 but in efferent lymph not until PIH 4, after the Igs had been brought to the lymph node from the tissue by the afferent lymph fluid. Thus, the content of IgA, IgM and IgG1 in efferent lymph appeared to be significantly influenced by that of afferent lymph during the inflammation, in the present study. This is further discussed later under "Modulation of immunoglobulins in the lymph node".

The concentration of the different Igs in tissue might to various extents have been influenced by active transport, selectively operating at the endothelium in the gland but not in the lymph node, or by local synthesis. It is, however, most likely that the increased tissue concentrations of IgA, IgM and IgG1 observed at PIH 2 were due to accumulation in the tissue, since the transfer further through the mammary epithelium of these Igs was hampered at this particular time, as shown in decreased epithelial CRs (Fig. 2C). It could, of course, also be speculated that the lower Ig concentrations in efferent lymph compared to those in afferent lymph at PIH 2 were due to suppressed endothelial transfer, selectively in the lymph node – rather than to increased concentrations in tissue. This is, however, not likely considering the particularly high permeability in the high endothelial venules in the lymph nodes.

Ig concentrations in *milk *increased more slowly than in any of the lymph fluids post-infusion, the highest values not being observed until PIH 4. In principal, the Igs in milk are substantially a result of transfer from the tissue fluid and it is therefore natural that the concentrations increase later in milk than in afferent lymph.

### The relative transfer of immunoglobulins

#### General comments

The results from this study show that, in general, the transfer of Ig through the *endothelium *is merely a result of diffusion while the transfer through the epithelium and the concentrations in milk is more influenced by selective mechanisms and local synthesis. Before endotoxin infusion the endothelial CR of each Ig was to various extents lower than that of BSA. The most plausible explanation is that the Ig molecules did not diffuse as easy through the tight junctions as BSA, under normal permeability conditions. Although there is a big variation in molecular size, each Ig is larger than the BSA molecule. IgM, being the largest molecule of the Igs, showed the lowest relative transfer through the endothelium which supports the speculation that the molecular size influences the transfer. During the inflammatory reaction the endothelial CR of each Ig increased to values almost equal to each other, however, still slightly lower than that of BSA.

The transfer of Igs through the mammary *epithelium *before as well as after the endotoxin infusion appeared to be highly affected by selective mechanisms or local production, IgG2 being the exception. The *milk:afferent lymph *CR for IgG1, IgM and IgA were notably higher than that of BSA at 0 h and for IgA the difference was huge. Additionally, during inflammation, the alterations of *milk:afferent lymph *CR for each of the three Igs were strikingly different from those of BSA.

#### IgG1

Previous studies, investigating blood and milk [3,32], have indicated that the concentration of IgG1 in milk is influenced by selective transport. The studies have, however, not made it possible to distinguish between the transfer through the endothelium and epithelium, respectively. Our results show that the selective mechanism is operating at the mammary epithelium, before as well as after the challenge. The epithelial CR of IgG1 in milk was higher than that of BSA, most pronounced at 0 h (Fig. 2C), while the endothelial CR of IgG1 and BSA were similar (Fig. 2A). IgG1 specific receptors located on the surface of alveolar epithelial cells have been identified in tissues from cows producing colostrum but never from cows in lactation [33-35]. Our observations show a selective mechanism being present in the transfer of IgG1 through the epithelium, also in lactating glands. The epithelial transfer of IgG1 appears to be more influenced by passive diffusion than that of IgA and IgM, since the relative IgG1 transfer increased from 0 h to PIH 4, along with increased epithelial permeability in the gland, in contrast to the transfer of IgA and IgM. However, a drop in *milk:afferent lymph *CR at PIH 2, similar to that of IgA and IgM, was observed also for IgG1 indicating a temporary suppression of the selective transport of IgG1 from tissue to milk at this time. A reduction of free IgG1 due to e.g. enhanced binding to leukocytes [36,37] is not a likely explanation since the cellular response in milk was barely detected, at this time.

#### IgA

In *milk*, the concentration of IgA was high, almost equal to that in afferent lymph before endotoxin infusion with a *milk:afferent lymph *CR of 0.97 to be compared to that of BSA of 0.02. These observations are in accordance with our previous studies where IgA concentration in milk was even higher than that in afferent lymph in the non-challenged mammary gland [5]. The results indicate a local synthesis of IgA in the mammary tissue, in addition to the amount of IgA diffusing from tissue fluid, in agreement with results from several previous scientific studies [25,38,39]. A local synthesis is further supported by the almost linear increase of IgA concentration in milk post-infusion (Fig. 1) in contrast to that of the other Igs which mainly occurred between PIH 2 and PIH 4. IgA producing plasmacells have been found in both infected and non-infected mammary parenchyma of lactating cows but mainly in the interalveolar stroma and only a few adjacent to the epithelial cells [25]. According to our results, IgA is released primarily into milk, not into tissue fluid, which suggests that the synthesis occur close to the epithelium rather than in deeper sub-epithelial tissues of the udder.

Interestingly, the relative concentration of IgA in milk compared to that in afferent lymph (the *milk:afferent lymph *CR) decreased from 0 h to PIH 4 (Fig. 2C) in spite of the gradually increasing epithelial permeability, diffusion of BSA and neutrophil influx to milk. The lowest CR was observed at PIH 2 indicating an inhibition mechanism at this time point. A similar pattern was observed for IgM (and IgG1). According to these observations the concentrations of IgA and IgM in milk during the inflammatory reaction were, on a whole, not substantially dependent on permeability conditions.

#### IgM

At 0 h, the mammary *endothelial *CR of IgM was *notably *lower than that of BSA (Fig. 2A). A similar relationship was observed between the CRs of IgM and BSA also at the endothelium in the *lymph node *(Fig. 2B). This indicates a factor that is limiting the diffusion of IgM compared to that of BSA. A plausible explanation is that the large size of the Ig M molecule makes passage through the capillary endothelium difficult under physiological permeability conditions. This is supported by the rapidly increased *afferent lymph:serum *and *efferent lymph:serum *CRs of IgM observed when the vascular permeability increased during inflammation. It is however, puzzling that the increase in concentration and CR of IgM was delayed in efferent lymph compared to afferent lymph since the passage of IgM through the endothelium of the highly permeable high endothelial venules in the lymph node should reasonably have occurred even more easily than in the mammary tissue capillaries. Apparently, the content of efferent lymph was strongly influenced by IgM brought there by the afferent lymph. This indicates a local source of IgM, in addition to blood, and/or an accumulation of IgM in the tissue due to hampered transfer over the mammary epithelium. In previous studies, IgM producing plasma cells have been observed in the mammary gland tissue but rarely close to the epithelial cells [25].

The highest CR of IgM at the mammary *epithelium *was, surprisingly, recorded at 0 h. In contrast to the endothelial CR, the epithelial CR of IgM tended to *decrease *after the endotoxin infusion. Even if the CR may be influenced by additional factors, it is notable that the increased epithelial permeability between tissue and milk, as shown in elevated BSA CR, was not reflected in the CR of IgM. The findings further indicate that the IgM transfer through the mammary epithelium was somehow hampered during this time of the inflammatory reaction. The underlying mechanism remains to be explained.

#### IgG2

Before endotoxin infusion the relative transfer of IgG2 through the *endothelium *to afferent lymph, as reflected in the *afferent lymph:serum *CR, was fairly similar to that of BSA, suggesting that the content of IgG2 in non-challenged afferent lymph is, in principal, a result of diffusion from blood only. This is in accordance with previous studies of IgG2 in milk compared to blood [3]. At PIH 2 the endothelial CR of IgG2 had increased, however, not at the same rate as that of BSA. Considering that the large influx of neutrophils to afferent lymph was observed at this time a possible explanation is that the amounts of free IgG2 was reduced due to enhanced binding to IgG2 specific surface receptors of the neutrophils [40].

The relative transfer of IgG2 from tissue into milk was almost identical to that of BSA before as well as during inflammation. Thus, the IgG2 transfer through the epithelium appears to be an effect of passive diffusion only.

### Modulation of immunoglobulins in the lymph node

It has been discussed to what extent the contents of efferent lymph reflect that of afferent lymph and how much the efferent lymph content is influenced by fluid and protein coming from blood through the postcapillary venules in the node. Igs in the local lymph node and efferent lymph are to some extent transferred from blood [8,30] and previous studies, focusing on protein in lymph, have reported that the blood derived contents may contribute 30–50% of the protein output in efferent lymph from un-stimulated lymph nodes [30,31]. However, this may vary between nodes in different regions. Since a significant function of the lymph node is to provide a meeting place for antigen-cell interactions in the initiation of the immune defence, it is quite obvious that the lymph formed also may be modulated within the node by targeted addition or trapping of Igs and Ig-producing cells [7,41] and additionally influenced by the content of afferent lymph flowing into the lymph node [8,30,31]. Thus, the degree of influence from different sources can be expected to vary dependent on whether the node is antigen stimulated or not.

In the present study concentration of each Ig isotype and BSA, respectively, was similar in the two lymph fluids, before the endotoxin challenge. This suggests, in agreement with the previous studies [30,31], that the content in both fluids were mainly blood derived at this time point. After challenge, Ig isotype concentrations in efferent lymph, particularly regarding IgA, IgM and IgG1, increased more slowly than in afferent lymph, in contrast to BSA, which increased equally rapid in both lymph fluids. These observations indicate that after the endotoxin challenge, Ig concentrations in efferent lymph were mainly influenced by the contents of the afferent lymph, flowing into the node and to a less extent dependent on transfer from blood.

## Conclusion

The most rapid increase of Igs was observed in afferent lymph, resulting in significantly higher concentration of each Ig isotype, except for IgG2, in afferent than in efferent lymph at PIH 2, contrary to before challenge. Ig concentrations in milk were in general lower than in lymph and they increased later. The transfer of Igs through the *endothelium *appeared to be merely a result of diffusion while the transfer through the *epithelium *and the Ig concentrations in milk seemed to be more influenced by selective mechanisms and local sources, respectively. In addition, the molecular size of the Igs appeared to negatively affect their transfer through the endothelium, particularly under normal permeability conditions when the CR of each Ig isotype, except for IgG2, was *lower *than that of BSA. However, at the mammary *epithelium *the opposite was observed; the CR of each Ig isotype, except for IgG2, was *higher *than that of BSA, before challenge. Additionally, the alterations in the epithelial CR of the Igs (IgG1, IgM and IgA) during inflammation were strikingly different from those of BSA. Our observations indicate a selective mechanism being present in the transfer of IgG1 through the epithelium, also in lactating glands which has not been previously shown. The results also indicate a local synthesis in the tissue of IgA and possibly also of IgM, released primarily into milk, not into tissue fluid suggesting that the synthesis occurs close to the epithelium. The IgG2 transfer through endothelium as well as epithelium appeared to be a result of passive diffusion only. In the lymph node, the content of efferent lymph was strongly influenced by IgG1, IgM and IgA brought to the node by the afferent lymph, from the mammary tissue and less dependent on transfer from blood.

## Competing interests

The authors declare that they have no competing interests.

## Authors' contributions

KÖ designed and planned the study and methods used, performed the surgery, oversaw and participated in the practical work and prepared the major part of the final manuscript, SL performed the sample collection and laboratory analyses, prepared a first draft of the manuscript and participated in preparing the final manuscript. Both authors read and approved the final manuscript.
